# Highly contiguous genome assembly of *Drosophila prolongata*—a model for evolution of sexual dimorphism and male-specific innovations

**DOI:** 10.1093/g3journal/jkae155

**Published:** 2024-07-13

**Authors:** David Luecke, Yige Luo, Halina Krzystek, Corbin Jones, Artyom Kopp

**Affiliations:** Department of Evolution and Ecology, University of California Davis, One Shields Ave, Davis, CA 95616, USA; USDA, ARS, PA, US Livestock Insects Research Lab, 2700 Fredericksburg Road, Kerrville, TX 78028, USA; Department of Evolution and Ecology, University of California Davis, One Shields Ave, Davis, CA 95616, USA; Biology Department of the University of North Carolina (UNC), 3159 Genome Sciences Building, 250 Bell Tower Drive, Chapel Hill, NC 27599, USA; Biology Department of the University of North Carolina (UNC), 3159 Genome Sciences Building, 250 Bell Tower Drive, Chapel Hill, NC 27599, USA; Department of Evolution and Ecology, University of California Davis, One Shields Ave, Davis, CA 95616, USA

**Keywords:** genome, Drosophila, sex dimorphism

## Abstract

*Drosophila prolongata* is a member of the *melanogaster* species group and *rhopaloa* subgroup native to the subtropical highlands of Southeast Asia. This species exhibits an array of recently evolved male-specific morphological, physiological, and behavioral traits that distinguish it from its closest relatives, making it an attractive model for studying the evolution of sexual dimorphism and testing theories of sexual selection. The lack of genomic resources has impeded the dissection of the molecular basis of sex-specific development and behavior in this species. To address this, we assembled the genome of *D. prolongata* using long-read sequencing and Hi–C scaffolding, resulting in a highly complete and contiguous (scaffold N50 2.2 Mb) genome assembly of 220 Mb. The repetitive content of the genome is 24.6%, the plurality of which are long terminal repeats retrotransposons (33.2%). Annotations based on RNA-seq data and homology to related species revealed a total of 19,330 genes, of which 16,170 are protein-coding. The assembly includes 98.5% of Diptera BUSCO genes, including 93.8% present as a single copy. Despite some likely regional duplications, the completeness of this genome suggests that it can be readily used for gene expression, genome-wide association studies (GWAS), and other genomic analyses.

## Introduction

**Fig. 1.**
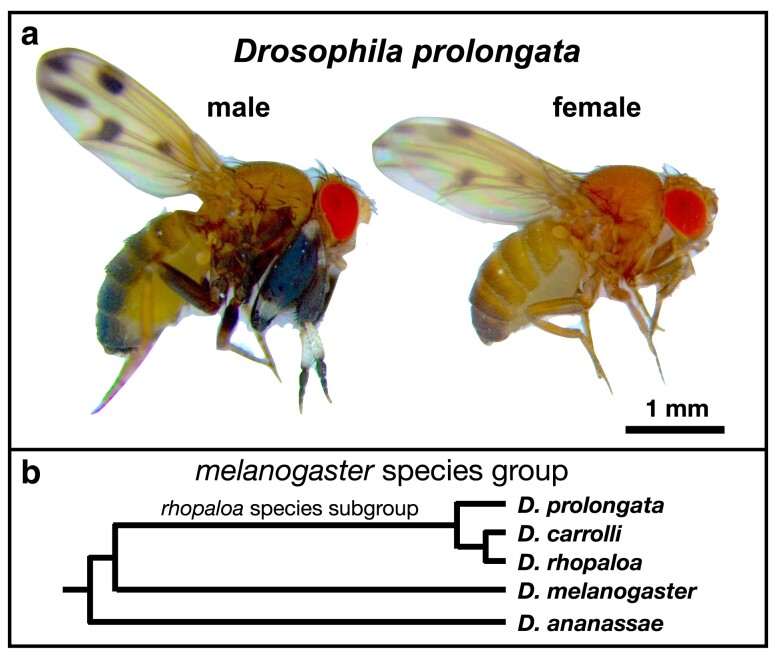
a) *Drosophila prolongata* has a suite of recently evolved male-specific traits, ideal for studying the evolution of sexual dimorphism. Most noticeable is the size and pigmentation banding of front legs in males. Other sexually dimorphic characteristics include wing spots, eye shape, pigmentation and increased length of second and third legs. b) Cladogram showing position of the *rhopaloa* species subgroup inside the *melanogaster* species group, with outgroup *D. ananassea* (RefSeq GCF_017639315.1).

*Drosophila prolongata* is a member of the *melanogaster* species group and *rhopaloa* subgroup native to Southeast Asia (Fig. 1b) (Singh and Gupta 1977; Toda 1991). The species has a suite of recently evolved male-specific morphological traits (Fig. 1a), including increased foreleg size, leg pigmentation, wing pigmentation, reversed sexual size dimorphism, and an expanded number of leg chemosensory organs (Luecke *et al*. 2022; Luecke and Kopp 2019; Luo *et al*. 2019). These traits are associated with derived behaviors, including male–male grappling and male leg vibration courtship displays, along with increased sexual dimorphism in cuticular hydrocarbon profiles (Amino and Matsuo 2023b, 2023a; Kudo *et al*. 2015, 2017; Luo *et al*. 2019; Setoguchi *et al*. 2014; Takau and Matsuo 2022; Toyoshima and Matsuo 2023).

The phylogenetic proximity to the model species *Drosophila melanogaster* and available genome sequences for closely related species *Drosophila rhopaloa* and *Drosophila carrolli* (Kim *et al*. 2021), which lack these derived traits, make this species a promising system to study the genetics of sexually dimorphic development, physiology, and behavior. A reference genome assembly and annotation for *D. prolongata* benefits such work as it would provide insight into the genomic evolutionary patterns associated with the evolution of the novel traits in *D. prolongata*. Presented here is a highly complete and contiguous assembly based on long-read Pacific Biosciences sequencing and Hi-C scaffolding, along with annotations for both *D. prolongata* and *D. carrolli* using *D. melanogaster* sequence homology and gene models based on RNA sequencing evidence and ab initio predictions.

## Materials and methods

### Genome line generation

The isofemale SaPa01 line and BaVi44 line were collected in SaPa and BaVi, Vietnam, respectively, by Dr Hisaki Takamori in September 2004. Virgin females were collected by isolating adults within 4 h of emergence. Four generations of full-sib matings were carried out to produce the genomic strain SaPa_ori_Rep25-2-1-1 (“Sapa_PacBio”). Fly strains were maintained at room temperature on standard cornmeal food provided by the UC Davis Fly Kitchen with filter paper for environment structure and pupariation substrate.

### Tissue collection

For genome assembly/scaffolding, adult male flies from the genome strain were moved onto nutrient-free agar media for at least 1 day to reduce microbial load, then collected into 1.5 mL tubes and flash-frozen in liquid nitrogen. Fifty frozen adult male individuals were sent on dry ice to Dovetail Genomics (Cantata Bio. LLC, dovetailgenomics.com) for DNA extraction, sequencing, and assembly. For gene expression data used in annotation, whole forelegs were dissected from carbon dioxide anesthetized males and females of the SaPa01 isofemale line, along with dissected heads from each sex of the genome strain.

### Sequencing and assembly

All genomic DNA extraction, sequencing, and assembly were carried out by Dovetail Genomics (Cantata Bio LLC, Scotts Valley, CA, USA). Genomic DNA was extracted with the Qiagen high molecular weight (HMW) genomic extraction kit (Qiagen, Germantown, MD, USA). DNA samples were quantified using a Qubit 2.0 Fluorometer (Life Technologies, Carlsbad, CA, USA). The PacBio SMRTbell library (∼20 kb) for PacBio Sequel was constructed using SMRTbell Express Template Prep Kit 2.0 (PacBio, Menlo Park, CA, USA) using the manufacturer-recommended protocol. The library was bound to polymerase using the Sequel II Binding Kit 2.0 (PacBio) and loaded onto PacBio Sequel II. Sequencing was performed on PacBio Sequel II 8 M single molecular real-time (SMRT) cells, generating 16 gigabases of data. An initial assembly based on 1.2 M PacBio reads was produced using FALCON (Chin *et al*. 2016) with Arrow polishing.

A Dovetail Hi-C library was prepared similarly as described previously (Lieberman-Aiden *et al*. 2009). Briefly, for each library, chromatin was fixed in place with formaldehyde in the nucleus and then extracted. Fixed chromatin was digested with DpnII, the 5′ overhangs filled in with biotinylated nucleotides, and free blunt ends were ligated subsequently. After ligation, crosslinks were reversed, and the DNA was purified from protein. Purified DNA was treated to remove biotin that was not internal to ligated fragments. The DNA was then sheared to ∼350 bp mean fragment size, and sequencing libraries were generated using NEBNext Ultra enzymes and Illumina-compatible adapters. Biotin-containing fragments were isolated using streptavidin beads before PCR enrichment of each library. The libraries were sequenced on an Illumina HiSeq X to a target depth of 30× coverage.

The input de novo assembly and Dovetail Hi-C library reads were used as input data for HiRise, a software pipeline designed specifically for using proximity ligation data to scaffold genome assemblies (Putnam *et al*. 2016). Dovetail Hi-C library sequences were aligned to the draft input assembly using a modified SNAP read mapper (http://snap.cs.berkeley.edu). The separations of Dovetail Hi-C read pairs mapped within draft scaffolds were analyzed by HiRise to produce a likelihood model for genomic distance between read pairs, and the model was used to identify and break putative misjoins, to score prospective joins, and make joins above a threshold. A second HiRise assembly was generated with additional Hi-C sequencing and the HiRise software pipeline.

RNA was extracted using TRIzol (Invitrogen, Waltham, MA, USA). For foreleg RNA, multiplexed stranded cDNA sequencing libraries were prepared using the NEBNext Ultra Directional RNA Library Prep Kit for Illumina (New England BioLabs, Ipswich, MA, USA) using poly(A) isolation magnetic beads. Libraries were sequenced on the Illumina HiSeq4000 platform by the UC Davis Genome Center. For head RNA, cDNA sequencing libraries were constructed using the TruSeq Stranded RNA Kit (Illumina, San Diego, CA, USA) and sequenced on the Illumina HiSeq4000 platform by Novogene (https://www.novogene.com/us-en/). Raw RNA-seq reads and assembled genome can be accessed with NCBI BioProject PRJNA1057277. Transcripts were assembled using Trinity 2.4.0 (Haas *et al*. 2013) with default options for stranded data.

### Gene prediction and annotation

Homology-based annotations for the *D. prolongata* and *D. carrolli* assemblies were generated using Liftoff 1.5.1 (Shumate and Salzberg 2021) with minimap2 2.17 (Li 2018) alignment based on the *D. melanogaster* GCF000001215.4 release 6 (Hoskins *et al*. 2015) *D. elegans* GCF000224195.1 2.0, and *D. rhopaloa* GCF000236305.1 2.0 (Kim *et al*. 2021) annotations downloaded from FlyBase (Gramates *et al*. 2022). Liftoff was run with the copies option and percent identity 0.80. Additional *D. prolongata* and *D. carrolli* gene models were inferred using MAKER 3.01.02 (Holt and Yandell 2011) with BLAST 2.11.0 (Camacho *et al*. 2009) and repeat masker 4.0.7, using EST evidence from the Trinity transcripts assembled based on foreleg and head RNA and protein homology evidence based on the combined protein sets from the *D. melanogaster* and *D. elegans* annotations also used for Liftoff. The annotations from different sources were then combined using gffcompare 10.4 (Pertea and Pertea 2020), genometools 1.5.9 (Gremme *et al*. 2013), and custom Python 3.7.6 scripts available at https://github.com/dluecke/annotation_tools.

### Removal of duplicate scaffolds

BUSCO (Manni *et al*. 2021) analysis of the Dovetail HiRise using the diptera_ocb10 lineage dataset revealed 200 complete but duplicated benchmark genes (Supplementary Table 1), indicating potential duplicated regions in the assembly. Scaffolds were assessed for BUSCO benchmark gene content and sorted by the percentage of duplicated BUSCO genes. Fifty three candidate scaffolds, ranging from 20,819 to 39,990,007 bp, contained at least one duplicated benchmark BUSCO gene (Supplementary Table 1). Inspection of MUMmer (Marçais *et al*. 2018) alignments between duplicate-containing candidates and scaffolds with alternate copies of the duplicated benchmark genes showed complete alignment across 27 of the candidate scaffolds (Supplementary Fig. 1). These 27 scaffolds (ranging from 20,819 to 541,551 bp) were considered fully duplicated and split from the assembly and annotation (Supplementary Table 1, files prolongataSaPa_WGS-RemovedDups.fa and prolongataSaPa_WGS-RemovedDups.gff in Dryad repository doi.org/10.5061/dryad.mpg4f4r6w) using SAMtools 1.15.1 (Li *et al*. 2009). Custom Python pandas 1.1.2 (McKinney 2010), and R 4.0.3 code for scaffold sorting by BUSCO scores, splitting assembly and annotation, and inspecting genome alignments are available at https://github.com/dluecke/annotation_tools.

### Identification of duplicate genes

The remaining duplicated genes in the *D. prolongata* deduplicated annotation were identified using reciprocal BLAST. Strand oriented regions corresponding to all “gene” features in both *D. prolonogata* and *D. rhopaloa* annotations were extracted from their respective assemblies using bedtools 2.29.2 (Quinlan and Hall 2010). *D. prolongata* gene regions were searched against all *D. prolongata* and all *D. rhopaloa* gene regions using blastn 2.14.1 (Camacho *et al*. 2009). BLAST results were combined and sorted by match alignment bit score, then duplicate status was assigned to pairs of *D. prolongata* genes if both regions had higher match scores with the corresponding *D. prolongata* region than to any gene region from *D. rhopaloa*. Custom Bash and Python scripts used in this process are available at https://github.com/dluecke/annotation_tools.

### Repeat analysis

Tandem repeats were annotated with Tandem Repeat Finder 4.09.1 (Benson 1999). A de novo library of classified repetitive element models was created using RepeatModeler 2.0 (Flynn *et al*. 2020). To reduce the run-to-run variations, repeat classification was based on five independent RepeatModeler runs with the following random seeds: 1681089287, 1687990919, 1683413925, 1683532158, and 1683532058. Custom R and Bash scripts are available at https://github.com/yige-luo/Repeat_analysis.

### Assembly and annotation evaluation

Assembly contiguity statistics were provided by Dovetail. Reference annotations *D. melanogaster* GCF_000001215.4 and *D. rhopaloa* GCF_018152115.1 were downloaded from the NCBI genomes database. Assembly completeness was assessed with BUSCO 5.3.2 (Manni *et al*. 2021) using the diptera_ocb10 lineage dataset, HMMER 3.1b2, and Mmseqs 5.34c21f2. Whole genome alignment between *D. prolongata* and *D. rhopaloa* assemblies was performed with MUMmer 4.0.0 (Marçais *et al*. 2018) using nucmer alignment with a minimum exact match 1,000 bp for alignment with *D. rhopaloa* and 500 bp for *D. melanogaster* alignment, and mummerplot plus custom Bash and R scripts (https://github.com/dluecke/annotation_tools) for visualization. Annotation statistics were found with genometools 1.5.9 (Gremme *et al*. 2013). Transcripts were extracted from annotations using gffread 0.9.12 (Pertea and Pertea 2020), and transcript completeness was assessed using the transcriptome mode of BUSCO.

## Results and discussion

### Assembly contiguity

**Fig. 2.**
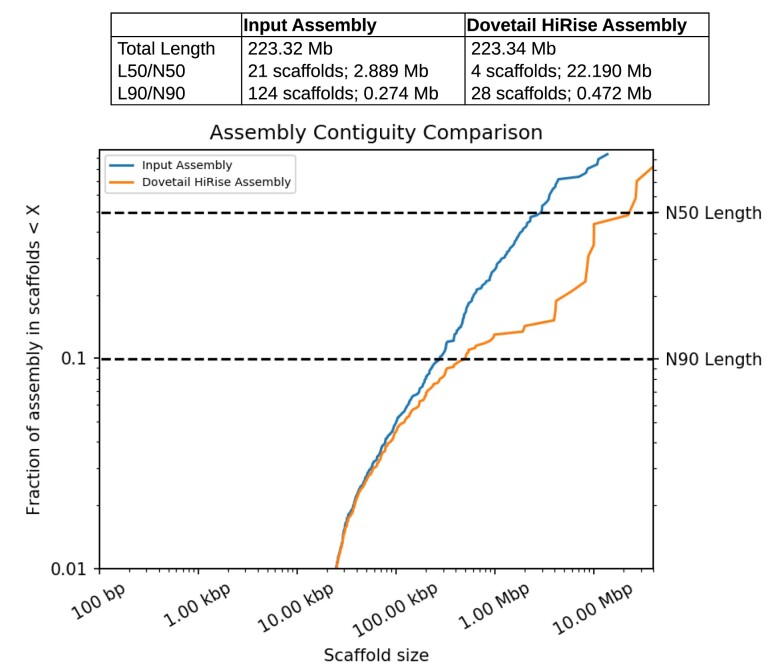
Dovetail assembly process generates high contiguity assembly. Comparison between initial PacBio FALCON with Arrow polished assembly (“Input Assembly”) and assembly generated by Dovetail Hi-C scaffolding method (“HiRise Assembly”), provided by Dovetail genomics. Each curve shows the fraction of the total length of the assembly in scaffolds of a given length or smaller. Horizontal dashed lines denote 50 and 90% of total assembly length; for the HiRise assembly 4 scaffolds 22.19 Mb and larger contain the majority of the assembly (L50 and N50) with 28 scaffolds containing more than 90% of the total length (L90), compared to 21 scaffolds 2.889 Mb and larger to cover half (and 124 scaffolds to cover 90%) of the input assembly. Scaffolds shorter than 1 kb are excluded.

**Fig. 3.**
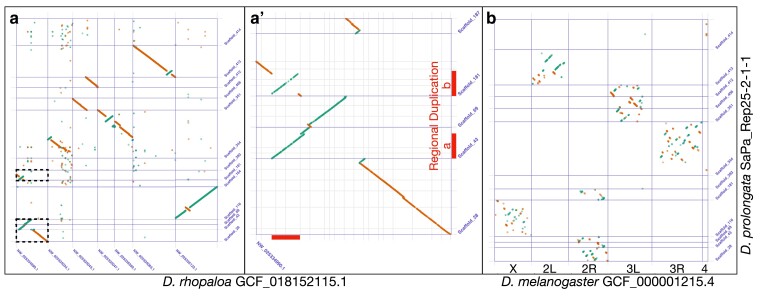
Whole genome alignments between major scaffolds of *D. prolongata* (>4 Mb, 84.0% of total size) assembly and reference assemblies. Sense matches are shown in green and cluster along positive slope lines, and antisense matches in orange clustering along negative slope lines. a) Alignment to *D. rhopaloa* reference based on minimum 1,000 bp matches, showing reference scaffolds >5 Mb (65.9% of total) as ordered in assembly; dashed boxed areas are expanded in panel A”. (a′) Zoom on portion of alignment A, showing regional duplication and inversion. Duplicated region is indicated by thick red segments parallel to axes. b) Alignment to major chromosome arms from *D. melanogaster* assembly (99.9% of total), based on minimum 500 bp matches. Large stretches of contiguity with limited large inversions are evident between *D. prolongata* and *D. rhopaloa* (a), while conservation of each chromosome arm's content along with considerable intra-arm rearrangement is seen between *D. prolongata* and *D. melanogaster* (b). A duplication spanning an inversion is evident between Scaffold_43 and Scaffold_181 (a’).

The Dovetail HiRise assembly scaffolding method (Fig. 2) followed by duplicate scaffold removal produced an assembly for *D. prolongata* with higher contiguity than the existing *D. rhopaloa* and *D. carrolli* assemblies, approaching the contiguity of the latest *D. melanogaster* reference (Table 1) as measured by N50. Whole genome alignments of the *D. prolongata* assembly to *D. rhopaloa* and *D. melanogaster* references (Fig. 3a) show long stretches of high identity with *D. rhopaloa* spanning nearly all large scaffolds.

**Table 1.** Statistics for assembly contiguity and completeness of the final (deduplicated) *D. prolongata* assembly alongside previously published *D. carrolli* GCA_018152295.1 assembly (Kim *et al*. 2021), reference assemblies *D. rhopaloa* GCF_018152115.1 and *D. melanogaster* GCF_000001215.4.

Assembly	*D. prolongata*	*D. carrolli*	*D. rhopaloa*	*D. melanogaster*
**Total length (bp)**	220,759,777	231,219,246	193,508,231	143,726,002
**Scaffolds**	387	338	228	1870
**N50 (bp)**	22,190,323	14,004,682	15,806,012	25,286,936
**L50**	4	5	5	3
**GC%**	40.11%	39.52%	39.87%	41.67%
**BUSCO complete, single copy**	93.7% (3,078)	97.8% (3,214)	98.1% (3,221)	98.5% (3,235)
**BUSCO complete, duplicated**	4.8% (158)	0.4% (13)	0.4% (12)	0.2% (8)
**BUSCO fragmented**	0.9% (29)	0.6% (19)	0.7% (24)	0.5% (16)
**BUSCO missing**	0.6% (20)	1.2% (39)	0.8% (28)	0.8% (26)

BUSCO statistics are for the 3,285 genes in the diptera_odb10 benchmark set.

### Assembly completeness

BUSCO results for assemblies (Table 1) show a comparable degree of completeness for the 3,285 genes in the BUSCO dipteran benchmark set between *D. prolongata* assembly and references, with 3,236 complete for *D. prolongata*, 3,233 complete for *D. rhopaloa*, and 3,243 complete for *D. melanogaster*. The whole genome alignments between the *D. prolongata* assembly and the *D. rhopaloa* (Fig. 3a) and *D. melanogaster* references (Fig. 3b) further show near complete highly contiguous coverage of the entire reference with regions of *D. prolongata* scaffolds, corresponding to all 5 major chromosome arms in the *D. melanogaster* genome. High completeness metrics for the final deduplicated assembly indicate the deduplication approach identified genuinely duplicated sequence.

### Repeat annotation

**Fig. 4.**
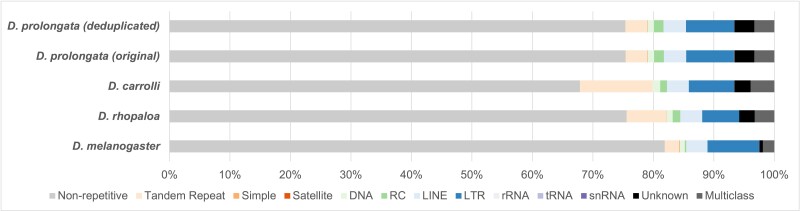
Genome-wide repeat content of *D. prolongata* (before and after deduplication) and related species. Repeat contents are color coded as follows. Low-complexity regions (Tandem repeats, simple repeats, satellite): orange palette, DNA transposons (DNA, RC): green palette, retrotransposons (LINE, LTR): blue palette, RNA: purple palette. Left-to-right order is consistent between legent and bar segments. Abbreviations for each repeat class are as follows. RC, rolling circle transposons; LINE, long interspersed nuclear element; LTR, long terminal repeats retrotransposon; snRNA: small-nuclear RNA; Unknown, unknown class of repeats/transposons; Multiclass, sequences belonging to more than one repeat class. Underlying values are reported in Table 2.

The *D. prolongata* genome exhibits a moderate level of repeat content (24.6%) comparable to the other species (Fig. 4). The vast majority (37/40) of classified repeat families are not specific to *D. prolongata*, except for two Long Interspersed Nuclear Element (LINE) retrotransposons (RTEs), RTE-BovB (Bovine-B) and L1 (LINE-1), and one DNA transposon, Crypton-V (Supplementary Table 2). We note, however, that further evidence is required to test whether these repeat families have evolved in *D. prolongata*, as all of them have only one identified member in one out of 5 RepeatModeler runs. Among the repetitive elements of *D. prolongata*, the most prominent repeat classes are Long Terminal Repeats retrotransposons (LTR, 32.2%), LINE (15.1%) and Tandem Repeats (14.6%, Table 2). A breakdown of repeat content by scaffolds across four species can be found in Supplementary Table 3.

**Table 2.** Repeat content of genome assemblies of *D. prolongata* and three reference species.

Repeat class	*D. prolongata* (%)	*D. carrolli* (%)	*D. rhopaloa* (%)	*D. melanogaster* (%)
Tandem repeat	3.627	12.003	6.601	2.421
Simple	0.008	0.007	0.008	0.007
Satellite	0.019	0.017	0.012	0.031
DNA	1.067	1.224	0.971	0.877
RC	1.595	1.122	1.274	0.218
LINE	3.727	3.626	3.612	3.526
LTR	7.939	7.505	6.141	8.525
rRNA	0.061	0.014	0.000	0.040
snRNA	0.000	0.001	0.004	0.000
tRNA	0.005	0.001	0.004	0.005
Unknown	3.276	2.686	2.519	0.575
Multiclass	3.311	3.926	3.260	1.896
**Total**	**24**.**636**	**32**.**131**	**24**.**406**	**18**.**121**

Compared with most long (>1 Mb) scaffolds, intermediate-sized scaffolds in *D. prolongata* assembly tend to show higher repeat content (Supplementary Figs. 2 and 3). Exceptions are found in scaffolds 414, scaffold 293, scaffold 164 and scaffold 280 (Supplementary Fig. 2), where LTR and LINE are overrepresented, reminiscent of the repeat profiles of several primary scaffolds in closely related species *D. carrolli* and *D. rhopaloa* (Supplementary Figs. 4 and 5), as well as the Y chromosome in *D. melanogaster* (Supplementary Fig. 6).

### Annotation completeness

Transcripts extracted from the annotation and assembly show that the *D. prolongata* and *D. carrolli* annotations have a high degree of completeness. However, they do not match the completeness of the *D. rhopaloa* and especially *D. melanogaster* references (Table 3), both in terms of gene inclusion and completeness of individual gene models, despite the higher number of protein-coding genes in the *D. prolongata* annotation (see “Potential regional duplications” section below). A higher number of BUSCO dipteran benchmark genes are missing in the *D. prolongata* (95) and *D. carrolli* (115) annotations compared to the *D. rhopaloa* (15) or *D. melanogaster* (0) references. Additionally, the transcripts in the *D. prolongata* and *D. carrolli* annotations are shorter than those from the references, and many more BUSCO dipteran benchmark genes are fragmented in the *D. prolongata* (109) and *D. carrolli* (89) annotations than for *D. rhopaloa* and D. *melanogaster* (both 3). These statistics show the limitations of current algorithmic annotation methods and indicate that care should be used when using gene models from these draft annotations. Despite these limitations, the overall completeness is quite high, with 93.8% of BUSCO benchmark genes covered in both *D. prolongata* and *D. carrolli* annotations (duplication scores are inflated by isoforms so are not reported), and comparable median transcript lengths in both. These gene models will provide a good foundation for future genetic studies in *D. prolongata* and relatives when used with the limitations of draft annotations in mind. Future iterations of the annotations, when informed by more transcriptome data, will improve gene model coverage and completeness.

**Table 3.** Statistics for annotation completeness for final (deduplicated) *D. prolongata* and *D. carrolli* annotations alongside reference annotations *D. rhopaloa* GCF_018152115.1 and *D. melanogaster* GCF_000001215.4.

Annotation	*D. prolongata*	*D. carrolli*	*D. rhopaloa*	*D. melanogaster*
**Genes**	19,330	16,346	15,463	17,559
**Protein-coding genes**	16,170	13,159	14,607	13,986
**Exons**	178,992	168,247	154,625	190,719
**Median transcript length (bp)**	1,635	1,758	1,995	1,954
**Longest transcript (bp)**	63,866	63,847	65,859	71,382
**BUSCO complete**	93.8% (3081)	93.8% (3081)	99.4% (3267)	99.9% (3282)
**BUSCO fragmented**	3.3% (109)	2.7% (89)	0.1% (3)	0.1% (3)
**BUSCO missing**	2.9% (95)	3.5% (115)	0.5% (15)	0.0% (0)

BUSCO duplication is not reported due to the conflation of isoform diversity and gene duplication.

### Potential regional duplications

The other major caveat for this assembly and annotation is the extent of identified duplication, even after removing duplicate scaffolds. This stands out most clearly in the *D. prolongata* assembly BUSCO scores, where 158 benchmark single-copy genes were identified as duplicated compared to 12 for *D. rhopaloa* and 8 for *D. melanogaster* (Table 1). Additional signals of duplicated regions include the total length of the draft assembly and total gene number in the annotation, which are both higher than in the *D. melanogaster* and *D. rhopaloa* references (Tables 1 and 3), and duplicated regions visible in the whole genome alignment (Fig. 3). This suggests some genome regions are represented more than once in the assembly, in addition to any true *D. prolongata*-specific duplication events. Our duplicate gene labeling method identified 945 of 19,330 genes (4.89%, close to the BUSCO duplicate frequency); these results are included in Supplementary Table 4, with a list of duplicated genes on Sheet 1 and the regions and relationships between pairs on Sheet 2; care should be taken when working with these genes and regions. We note that all major (>1 Mb) scaffolds in *D. prolongata* have duplicated BUSCO genes even after removal of the fully duplicate scaffolds (Supplementary Table 1, Supplementary Fig. 3). In contrast, removed scaffolds tend to be intermediate in size and have less repeat content (Supplementary Fig. 7). Remaining BUSCO duplications per scaffold for the final assembly are provided in Sheet 2 of Supplementary Table 1.

Duplication artifacts often result from heterozygosity persisting through inbreeding (Guo *et al*. 2016; Kardos *et al*. 2018; Smith *et al*. 2019). Segregating inversions, in particular, can capture stretches of heterozygosity and cause the assembler to split haplotypes into separate scaffolds. Consistent with this explanation, the largest remaining duplication candidate visible in the whole genome alignment spans a segregating inversion (Fig. 3a′). Sorting biologically real from artefactual duplicates is a key area of improvement for future *D. prolongata* assemblies.

## Supplementary Material

jkae155_Supplementary_Data

## Data Availability

The final deduplicated assembly for this Whole Genome Shotgun project has been deposited at DDBJ/ENA/GenBank under the accession JAYMZC000000000; the version described in this paper is version JAYMZC010000000. All sequence data used for genome annotation have been deposited in the NCBI Sequence Read Archive under BioProject PRJNA1057277. Genome annotation files for *D. prolongata* and *D. carrolli*, the Dovetail Falcon and HiRise assemblies (containing duplicate scaffolds), sequence file for removed duplicate scaffolds, and all sequence and information files provided by Dovetail have been uploaded to Dryad (doi.org/10.5061/dryad.mpg4f4r6w). All custom scripts are available at https://github.com/dluecke/annotation_tools and https://github.com/yige-luo/Repeat_analysis. Supplemental material available at G3 online.

## References

[jkae155-B1] Amino K, Matsuo T. 2023a. Effects of a past contest on the future winning probability in a hyper-aggressive fruit fly. Ethology. 129(8):380–389. doi:10.1111/eth.13375.

[jkae155-B2] Amino K, Matsuo T. 2023b. Reproductive advantage of the winners of male-male competition in *Drosophila Prolongata*. Behav Processes. 206:104831. doi:10.1016/j.beproc.2023.104831. 36693576

[jkae155-B3] Benson G . 1999. Tandem repeats finder: a program to analyze DNA sequences. Nucleic Acids Res. 27(2):573–580. doi:10.1093/nar/27.2.573. 9862982 PMC148217

[jkae155-B4] Camacho C, Coulouris G, Avagyan V, Ma N, Papadopoulos J, Bealer K, Madden TL. 2009. BLAST+: architecture and applications. BMC Bioinformatics. 10:421. doi:10.1186/1471-2105-10-421. 20003500 PMC2803857

[jkae155-B5] Chin CS, Peluso P, Sedlazeck FJ, Nattestad M, Concepcion GT, Clum A, Dunn C, O'Malley R, Figueroa-Balderas R, Morales-Cruz A, et al 2016. Phased diploid genome assembly with single-molecule real-time sequencing. Nat Methods. 13(12):1050–1054. doi:10.1038/nmeth.4035. 27749838 PMC5503144

[jkae155-B6] Flynn JM, Hubley R, Goubert C, Rosen J, Clark AG, Feschotte C, Smit AF. 2020. RepeatModeler2 for automated genomic discovery of transposable element families. Proc Natl Acad Sci U S A. 117(17):9451–9457. doi:10.1073/pnas.1921046117. 32300014 PMC7196820

[jkae155-B7] Gramates LS, Agapite J, Attrill H, Calvi BR, Crosby MA, Dos Santos G, Goodman JL, Goutte-Gattat D, Jenkins VK, Kaufman T, et al 2022. FlyBase: a guided tour of highlighted features. Genetics. 220(4):iyac035. doi:10.1093/genetics/iyac035. 35266522 PMC8982030

[jkae155-B8] Gremme G, Steinbiss S, Kurtz S. 2013. Genome tools: a comprehensive software library for efficient processing of structured genome annotations. IEEE/ACM Trans Comput Biol Bioinform. 10(3):645–656. doi:10.1109/TCBB.2013.68. 24091398

[jkae155-B9] Guo L, Zhang S, Rubinstein B, Ross E, Alvarado AS. 2016. Widespread maintenance of genome heterozygosity in *Schmidtea mediterranea*. Nat Ecol Evol. 1(1):19. doi:10.1038/s41559-016-0019. 28812561 PMC5556695

[jkae155-B10] Haas BJ, Papanicolaou A, Yassour M, Grabherr M, Philip D, Bowden J, Couger MB, Eccles D, Li B, Lieber M, et al 2013. De Novo transcript sequence recostruction from RNA-Seq: reference generation and analysis with Trinity. Nat Protoc. 8(8):1494–1512. doi:10.1038/nprot.2013.084. 23845962 PMC3875132

[jkae155-B11] Holt C, Yandell M. 2011. MAKER2: an annotation pipeline and genome-database management tool for second-generation genome projects. BMC Bioinformatics. 12:491. doi:10.1186/1471-2105-12-491. 22192575 PMC3280279

[jkae155-B12] Hoskins RA, Carlson JW, Wan KH, Park S, Mendez I, Galle SE, Booth BW, Pfeiffer BD, George RA, Svirskas R, et al 2015. The release 6 reference sequence of the Drosophila melanogaster genome. Genome Res. 25(3):445–458. doi:10.1101/gr.185579.114. 25589440 PMC4352887

[jkae155-B13] Kardos M, Åkesson M, Fountain T, Flagstad Ø, Liberg O, Olason P, Sand H, Wabakken P, Wikenros C, Ellegren H. 2018. Genomic consequences of intensive inbreeding in an isolated wolf population. Nat Ecol Evol. 2(1):124–131. doi:10.1038/s41559-017-0375-4. 29158554

[jkae155-B14] Kim BY, Wang JR, Miller DE, Barmina O, Delaney E, Thompson A, Comeault AA, Peede D, D'Agostino ERR, Pelaez J, et al 2021. Highly contiguous assemblies of 101 drosophilid genomes. Elife. 10:e66405. doi:10.7554/eLife.66405. 34279216 PMC8337076

[jkae155-B15] Kudo A, Shigenobu S, Kadota K, Nozawa M, Shibata TF, Ishikawa Y, Matsuo T. 2017. Comparative analysis of the brain transcriptome in a hyper-aggressive fruit fly, *Drosophila Prolongata*. Insect Biochem Mol Biol. 82:11–20. doi:10.1016/j.ibmb.2017.01.006. 28115271

[jkae155-B16] Kudo A, Takamori H, Watabe H, Ishikawa Y, Matsuo T. 2015. Variation in morphological and behavioral traits among isofemale strains of *Drosophila Prolongata* (Diptera: drosophilidae). Entomol Sci. 18(2):221–229. doi:10.1111/ens.12116.

[jkae155-B17] Li H . 2018. Minimap2: pairwise alignment for nucleotide sequences. Bioinformatics. 34(18):3094–3100. doi:10.1093/bioinformatics/bty191. 29750242 PMC6137996

[jkae155-B18] Li H, Handsaker B, Wysoker A, Fennell T, Ruan J, Homer N, Marth G, Abecasis G, Durbin R. 2009. The sequence alignment/map format and SAMtools. Bioinformatics. 25(16):2078–2079. doi:10.1093/bioinformatics/btp352. 19505943 PMC2723002

[jkae155-B19] Lieberman-Aiden E, Van Berkum NL, Williams L, Imakaev M, Ragoczy T, Telling A, Amit I, Lajoie BR, Sabo PJ, Dorschner MO, et al 2009. Comprehensive mapping of long-range interactions reveals folding principles of the human genome. Science. 326(5950):289–293. doi:10.1126/science.1181369. 19815776 PMC2858594

[jkae155-B20] Luecke D, Kopp A. 2019. Sex-Specific evolution of relative leg size in *Drosophila Prolongata* results from changes in the intersegmental coordination of tissue growth. Evolution. 73(11):2281–2294. doi:10.1111/evo.13847. 31595502 PMC6834887

[jkae155-B21] Luecke D, Rice G, Kopp A. 2022. Sex-Specific evolution of a *Drosophila* sensory system via interacting *Cis*- and *Trans*-regulatory changes. Evol Dev. 24(1–2):37–60. doi:10.1111/ede.12398. 35239254 PMC9179014

[jkae155-B22] Luo Y, Zhang Y, Farine JP, Ferveur JF, Ramírez S, Kopp A. 2019. Evolution of sexually dimorphic pheromone profiles coincides with increased number of male-specific chemosensory organs in *Drosophila prolongata*. Ecol Evol. 9(23):13608–13618. doi:10.1002/ece3.5819. 31871670 PMC6912897

[jkae155-B23] Manni M, Berkeley MR, Seppey M, Simão FA, Zdobnov EM. 2021. BUSCO update: novel and streamlined workflows along with broader and deeper phylogenetic coverage for scoring of eukaryotic, prokaryotic, and viral genomes. Mol Biol Evol. 38(10):4647–4654. doi:10.1093/molbev/msab199. 34320186 PMC8476166

[jkae155-B24] Marçais G, Delcher AL, Phillippy AM, Coston R, Salzberg SL, Zimin A. 2018. MUMmer4: a fast and versatile genome alignment system. PLoS Comput Biol. 14(1):e1005944. doi:10.1371/journal.pcbi.1005944. 29373581 PMC5802927

[jkae155-B25] McKinney W . 2010. Data structures for statistical computing in python. Proceedings of the 9th Python in Science Conference (1):56–61.

[jkae155-B26] Pertea G, Pertea M. 2020. GFF utilities: GffRead and GffCompare [Version 2; Peer Review: 3 Approved]. F1000Res. 9:ISCB Comm J-304. doi:10.12688/f1000research.23297.2. PMC7222033 32489650

[jkae155-B27] Putnam NH, O'Connell BL, Stites JC, Rice BJ, Blanchette M, Calef R, Troll CJ, Fields A, Hartley PD, Sugnet CW, et al 2016. Chromosome-scale shotgun assembly using an in vitro method for long-range linkage. Genome Res. 26(3):342–350. doi:10.1101/gr.193474.115. 26848124 PMC4772016

[jkae155-B28] Quinlan AR, Hall IM. 2010. BEDTools: a flexible suite of utilities for comparing genomic features. Bioinformatics. 26(6):841–842. doi:10.1093/bioinformatics/btq033. 20110278 PMC2832824

[jkae155-B29] Setoguchi S, Takamori H, Aotsuka T, Sese J, Ishikawa Y, Matsuo T. 2014. Sexual dimorphism and courtship behavior in *Drosophila prolongata*. J Ethol. 32(2):91–102. doi:10.1007/s10164-014-0399-z.

[jkae155-B30] Shumate A, Salzberg SL. 2021. Liftoff: accurate mapping of gene annotations. Bioinformatics. 37(12):1639–1643. doi:10.1093/bioinformatics/btaa1016. 33320174 PMC8289374

[jkae155-B31] Singh BK, Gupta JP. 1977. Two new and two unrecorded Species of the genus *Drosophila* fallen (Diptera: Drosophilidae) from Shillong, Meghalaya, India. Proc Zool Soc (Calcutta). 30:31–38.

[jkae155-B32] Smith NMA, Wade C, Allsopp MH, Harpur BA, Zayed A, Rose SA, Engelstädter J, Chapman NC, Yagound B, Oldroyd BP. 2019. Strikingly high levels of heterozygosity despite 20 years of inbreeding in a clonal honey bee. J Evol Biol. 32(2):144–152. doi:10.1111/jeb.13397. 30414283

[jkae155-B33] Takau A, Matsuo T. 2022. Contribution of visual stimuli to mating and fighting behaviors of *Drosophila prolongata*. Entomol Sci. 25(4):e12529. doi:10.1111/ens.12529.

[jkae155-B34] Toda MJ . 1991. Drosophilidae (Diptera) in Myanmar (Burma) VII. The *Drosophila melanogaster* Species-group, excepting the *D. Montium* Species-subgroup. Orient Insects. 25(1):69–94. doi:10.1080/00305316.1991.10432216.

[jkae155-B35] Toyoshima N, Matsuo T. 2023. Fight outcome influences male mating success in *Drosophila prolongata*. J Ethol. 41(2):119–127. doi:10.1007/s10164-023-00778-1.

