# Correction to: Highly contiguous genome assembly of *Drosophila prolongata*—a model for evolution of sexual dimorphism and male-specific innovations

**DOI:** 10.1093/g3journal/jkae253

**Published:** 2024-11-07

This is a correction to: David Luecke, Yige Luo, Halina Krzystek, Corbin Jones, Artyom Kopp, Highly contiguous genome assembly of *Drosophila prolongata*—a model for evolution of sexual dimorphism and male-specific innovations, *G3 Genes|Genomes|Genetics*, Volume 14, Issue 10, October 2024, jkae155, https://doi.org/10.1093/g3journal/jkae155

In the originally published version of the manuscript, there was a failure to acknowledge the equal contribution of the first two authors. The Acknowledgment section of the article has thus been amended to read additionally: “Dr David Luecke and Dr Yige Luo contributed equally to this paper.”

