# Supplementary material for: Highly contiguous genome assembly of Drosophila prolongata—a model for evolution of sexual dimorphism and male-specific innovations
Source: G3 (Bethesda). 2024 Jul 13;14(10):jkae155. doi: 10.1093/g3journal/jkae155 (PMC11457088; doi:10.1093/g3journal/jkae155)
Supplement: jkae155_Supplementary_Data [file jkae155_supplementary_data.zip › Figure_S3_G3-2024-405153.pdf]

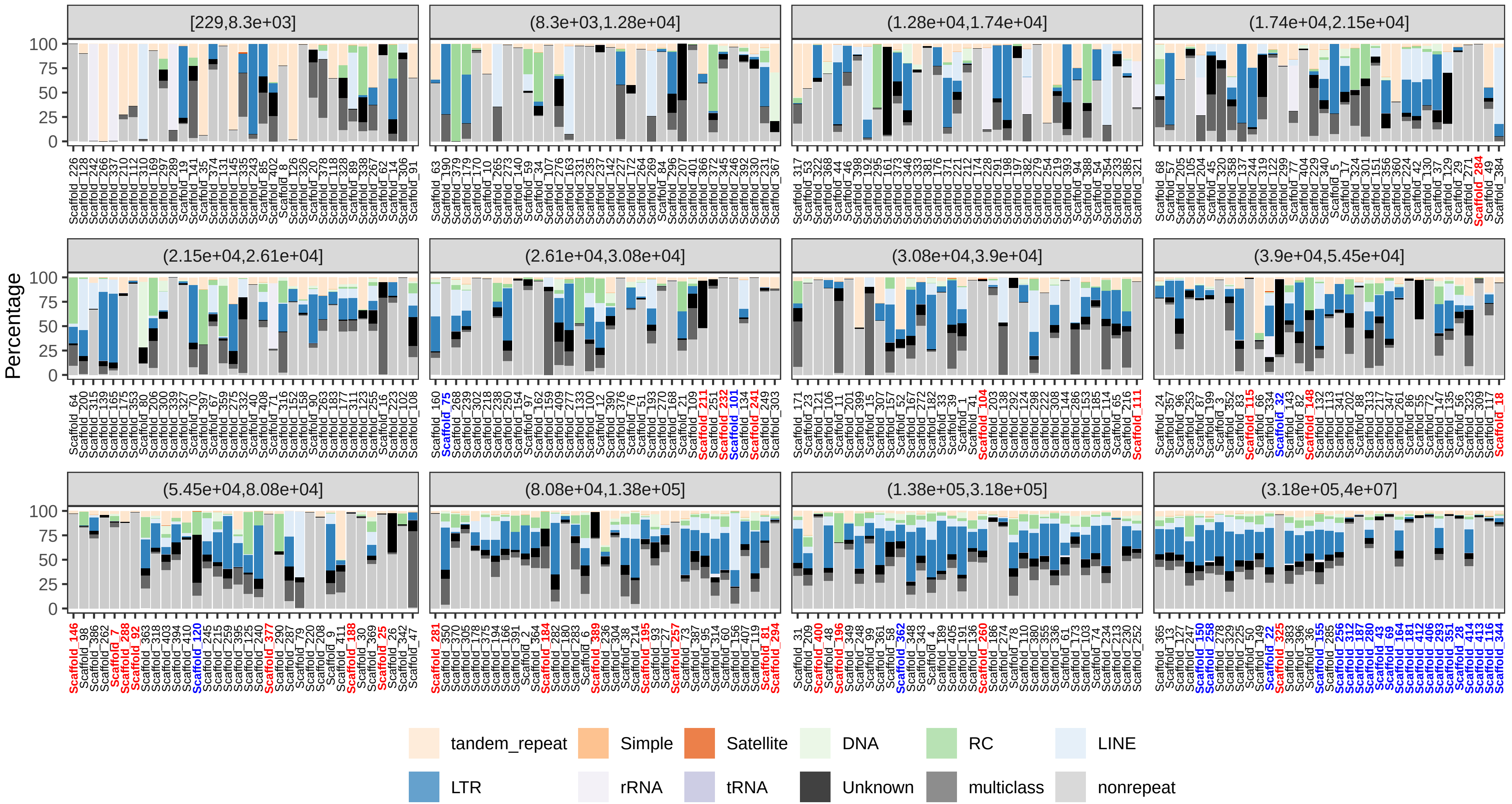
[229,8.3e+03]
(8.3e+03,1.28e+04]
(1.28e+04,1.74e+04]
(1.74e+04,2.15e+04]
(2.15e+04,2.61e+04]
(2.61e+04,3.08e+04]
(3.08e+04,3.9e+04]
(3.9e+04,5.45e+04]
(5.45e+04,8.08e+04]
(8.08e+04,1.38e+05]
(1.38e+05,3.18e+05]
(3.18e+05,4e+07]
Percentage
0
25
50
75
100
tandem_repeat
Simple
Satellite
DNA
RC
LINE
LTR
rRNA
tRNA
Unknown
multiclass
nonrepeat
