# Supplementary figures and images for: Highly contiguous genome assembly of Drosophila prolongata—a model for evolution of sexual dimorphism and male-specific innovations
Source: G3 (Bethesda). 2024 Jul 13;14(10):jkae155. doi: 10.1093/g3journal/jkae155 (PMC11457088; doi:10.1093/g3journal/jkae155)

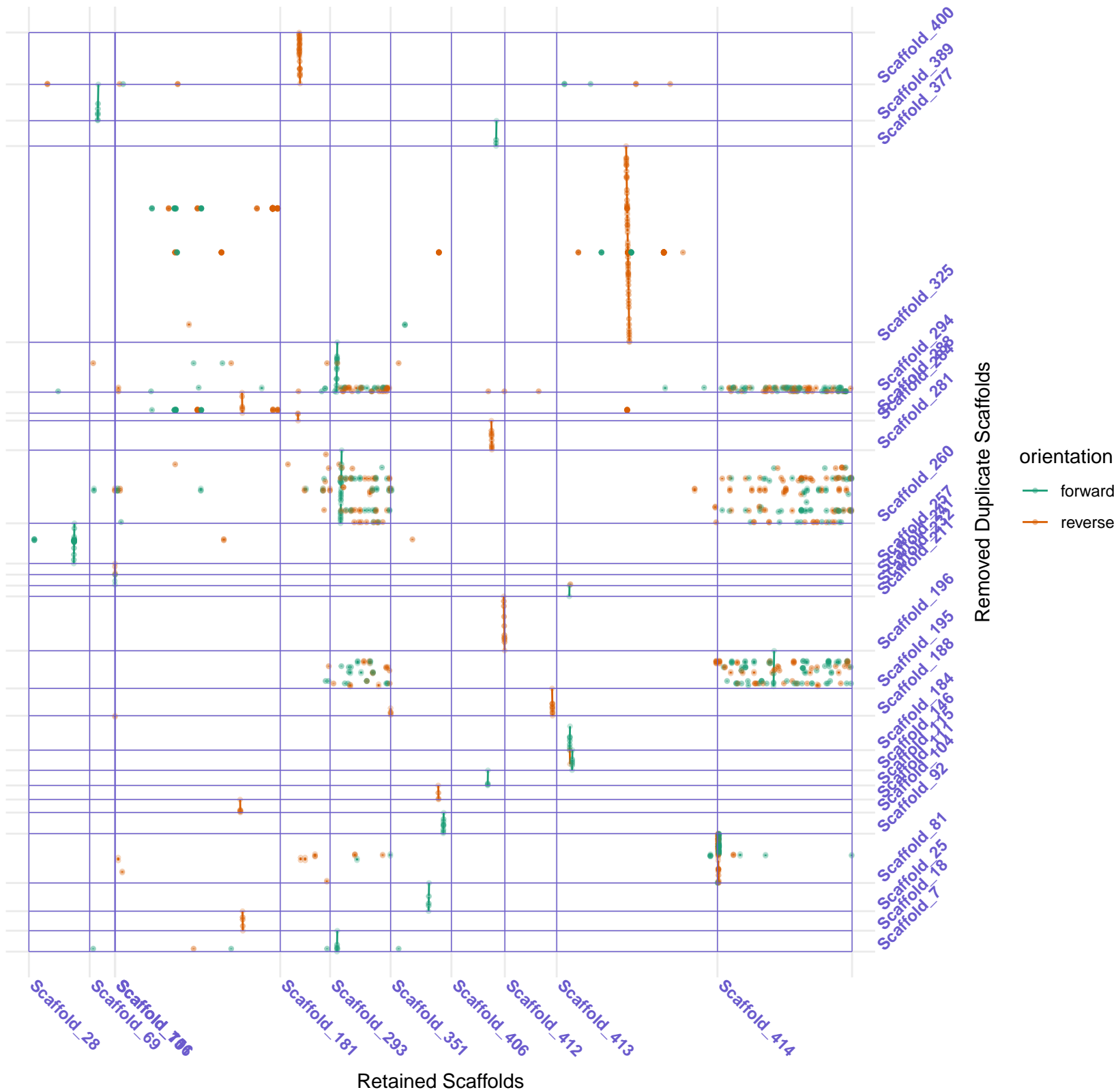

Supplement: jkae155_Supplementary_Data [file jkae155_supplementary_data.zip › Figure_S1_G3-2024-405153.pdf]

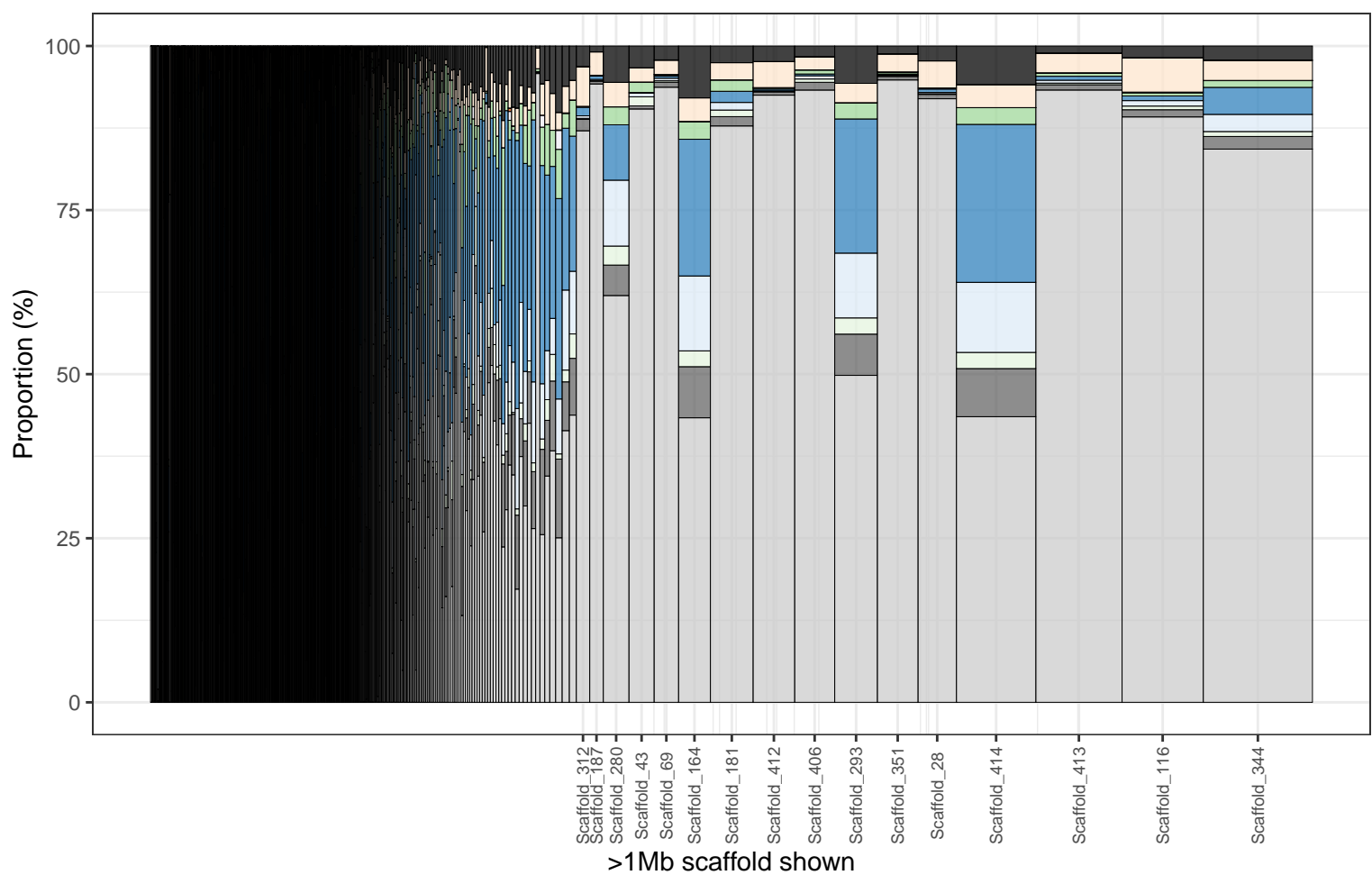

Supplement: jkae155_Supplementary_Data [file jkae155_supplementary_data.zip › Figure_S2_G3-2024-405153.pdf]

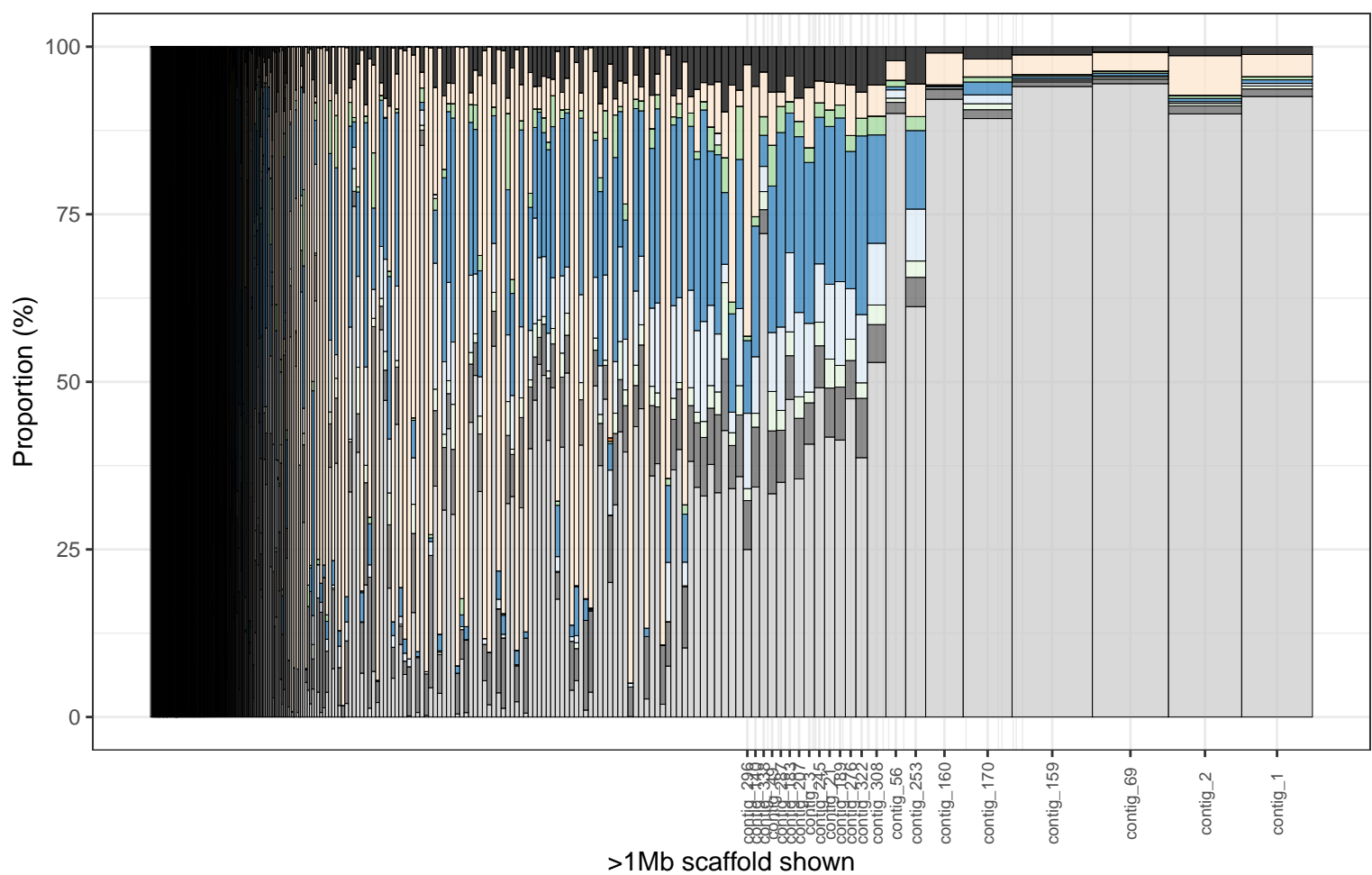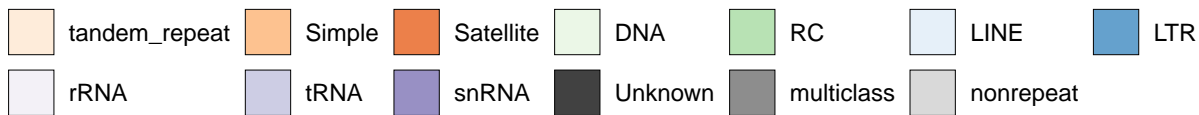

Supplement: jkae155_Supplementary_Data [file jkae155_supplementary_data.zip › Figure_S4_G3-2024-405153.pdf]

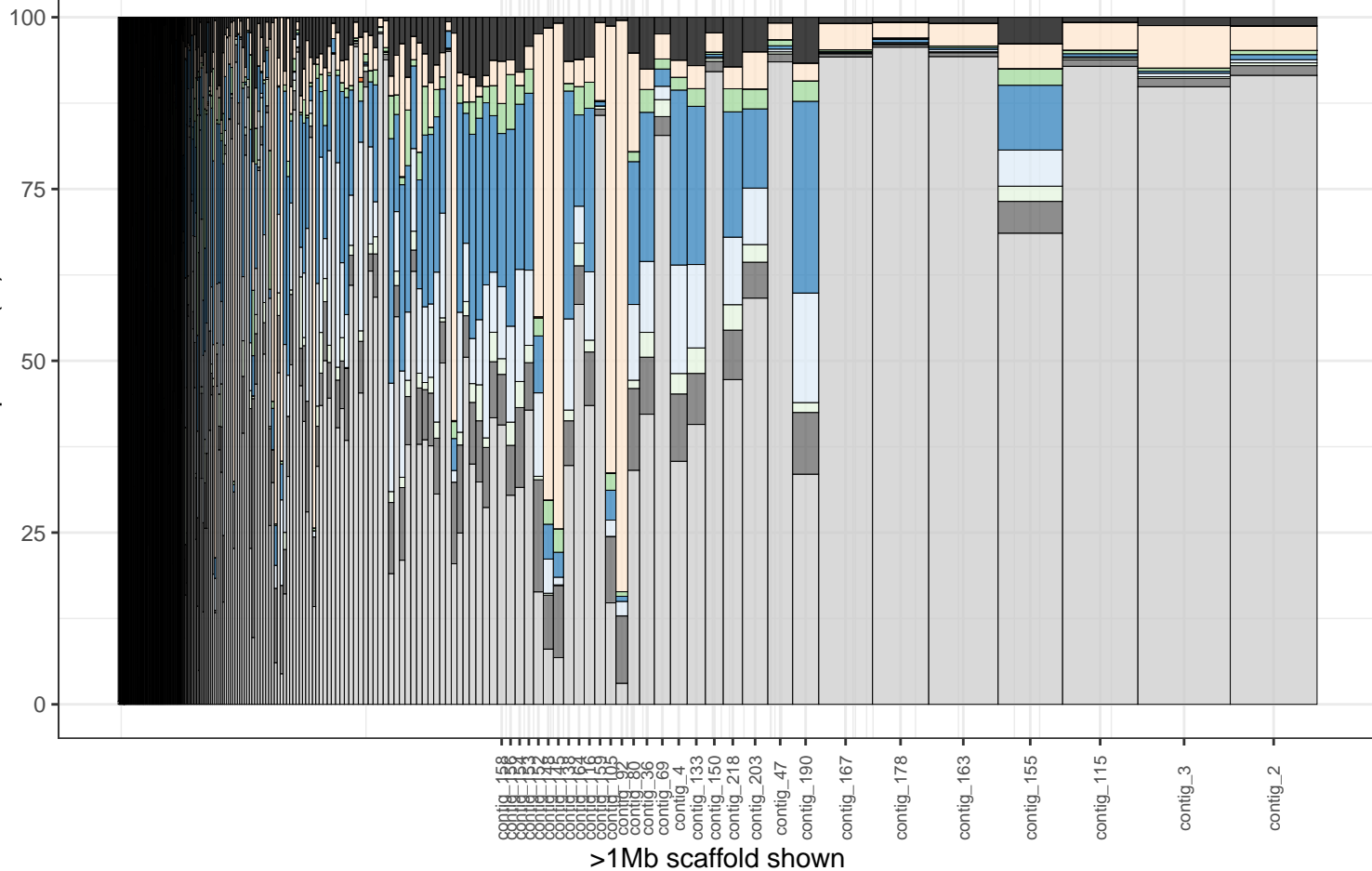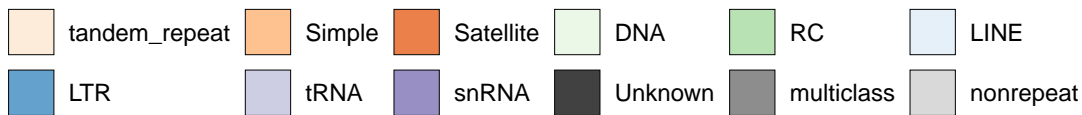

Supplement: jkae155_Supplementary_Data [file jkae155_supplementary_data.zip › Figure_S5_G3-2024-405153.pdf]

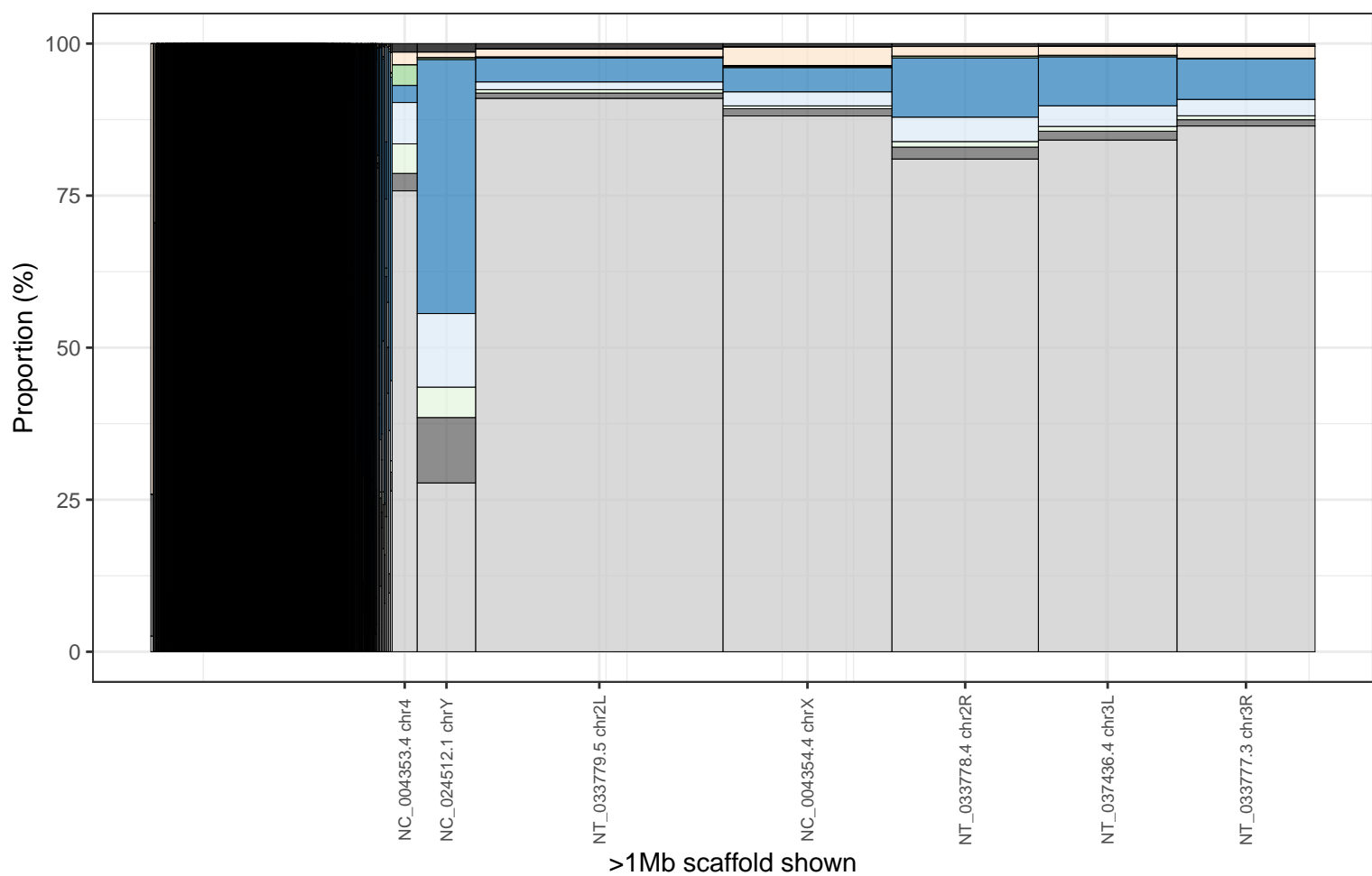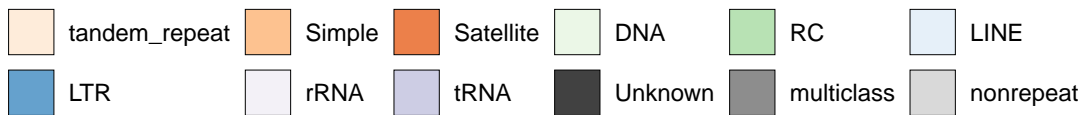

Supplement: jkae155_Supplementary_Data [file jkae155_supplementary_data.zip › Figure_S6_G3-2024-405153.pdf]

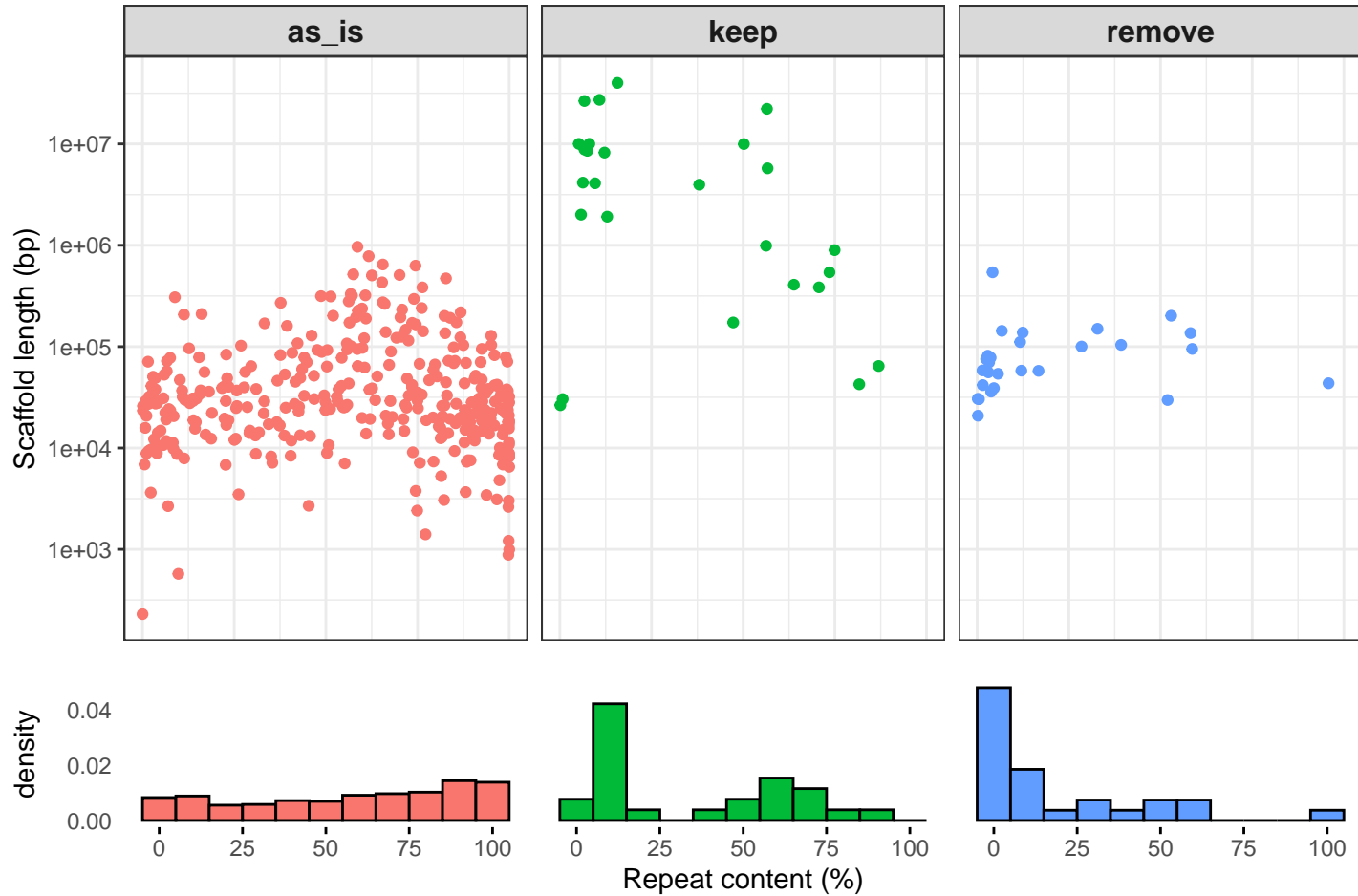

Supplement: jkae155_Supplementary_Data [file jkae155_supplementary_data.zip › Figure_S7_G3-2024-405153.pdf]
